# Gut microbiota characteristics in neonatal respiratory distress syndrome and the therapeutic potential of probiotics in recovery

**DOI:** 10.3389/fmicb.2025.1544055

**Published:** 2025-04-04

**Authors:** Yongcheng Fu, Xiujuan Wang, Lintao Nie, Zhaorui Wang, Xiao Ma, Lijia Wu, Liping Han, Wenjun Fu, Ruoming Wang, Hongyan Ren, Da Zhang, Juan Ding

**Affiliations:** ^1^Department of Pediatric Surgery, The First Affiliated Hospital of Zhengzhou University, Zhengzhou, China; ^2^Department of Nursing, The First Affiliated Hospital of Zhengzhou University, Zhengzhou, China; ^3^Translational Medicine Research Center, The Fifth Clinical Medical College of Henan University of Chinese Medicine (Zhengzhou People's Hospital), Zhengzhou, China; ^4^Department of Human Resources, The First Affiliated Hospital of Zhengzhou University, Zhengzhou, China; ^5^Department of Neonatal Intensive Care Unit, The First Affiliated Hospital of Zhengzhou University, Zhengzhou, China; ^6^Department of Gynecology, The First Affiliated Hospital of Zhengzhou University, Zhengzhou, China; ^7^Department of Obstetrics, The First Affiliated Hospital of Zhengzhou University, Zhengzhou, China; ^8^Shanghai Mobio Biomedical Technology Co., Ltd., Shanghai, China

**Keywords:** 16s rRNA sequencing, neonatal respiratory distress syndrome, gut microbiota, probiotics, preterm infants, predictive diagnosis

## Abstract

**Background:**

Neonatal Respiratory Distress Syndrome (NRDS) is a common and severe respiratory disorder in neonates, particularly among preterm infants (PTIs), and is often associated with hypoxemia and multiple organ dysfunction. This study aims to investigate the gut microbiota characteristics in NRDS and the potential regulatory role of probiotics in restoring gut microbiota dysbiosis.

**Methods:**

This study enrolled 55 PTIs diagnosed with NRDS and 26 preterm infants without NRDS. The NRDS group was classified into two groups based on treatment: an antibiotic-only group (TA group, *N* = 30) and an antibiotic plus probiotics group (TB group, *N* = 25). Fecal samples were collected within 48 h of birth and again after recovery, for 16S rRNA sequencing.

**Results:**

The study revealed that the gut microbiota diversity in the NRDS group was significantly greater than in the non-NRDS group, and the microbiota composition in the NRDS group was closely associated with multiple clinical indicators, including Apgar score, pH, PaO_2_, and PaCO_2_. Notably, the abundance of bacteria such as *Muribaculaceae Incertae Sedis*, *Rhodococcus*, and *Corynebacterium* was significantly higher in the NRDS group, which may contribute to disease progression. ROC analysis suggested that gut microbiota could serve as potential biomarkers for diagnosing NRDS. Probiotic intervention notably restored the gut microbiota structure in the NRDS group, particularly by enhancing the abundance of beneficial genera such as *Streptococcus*, *Bifidobacterium*, and *Clostridium*. This intervention reduced the microbiota disparity between the NRDS group and normal one-month-old children, thereby slowing disease progression.

**Conclusion:**

This study demonstrated that the NRDS displayed an increase in gut microbiota diversity and alterations in specific bacterial populations, both of which were closely correlated with clinical data. Probiotic treatment aids in restoring the disrupted gut microbiota in NRDS infants, promoting disease recovery, and providing new biomarkers and clinical strategies for managing NRDS.

## Introduction

Neonatal Respiratory Distress Syndrome (NRDS) is a common and serious respiratory disorder, particularly prevalent in PTIs (Dani et al., 1999; Gallacher et al., 2016). The pathogenesis of NRDS is primarily associated with the underdevelopment of the lungs in PTIs and the deficiency of pulmonary surfactant (PS) (Hermansen and Lorah, 2007). PS is a lipoprotein complex secreted by type II alveolar cells. It reduces surface tension in the alveoli, preventing their collapse (Possmayer et al., 2023). In PTIs, however, inadequate synthesis and secretion of PS hinder alveolar expansion, resulting in alveolar collapse and impaired ventilation (Jobe and Ikegami, 2000). NRDS-induced hypoxemia and carbon dioxide retention significantly impair respiratory function and may lead to systemic complications, such as acidosis, cardiovascular instability, and multi-organ dysfunction (Liu et al., 2010). As the disease progresses, NRDS may lead to long-term respiratory sequelae and increase the risk of neonatal mortality.

The gut-lung axis plays a crucial role in neonatal respiratory diseases, such as bronchopulmonary dysplasia and infant cystic fibrosis (Gallacher et al., 2020; Frayman et al., 2024). The gut-lung axis describes the mechanism by which the lungs and the gut interact through microbiota, their metabolites, and immune signals (Perdijk et al., 2024). However, research on the impact of the gut-lung axis on NRDS remains limited. Dysbiosis has been linked to various neonatal diseases, such as necrotizing enterocolitis (NEC), asthma, and pediatric respiratory infections (Fujimura et al., 2016; McDonnell et al., 2021; Willis and Ambalavanan, 2021). Dysbiosis leads to a reduction in beneficial bacteria and an overgrowth of harmful bacteria, compromising gut barrier function, increasing inflammation, and disrupting immune regulation (Hou et al., 2022). In PTIs and NRDS patients, interventions such as antibiotics and mechanical ventilation may disrupt the gut microbiota, potentially exacerbating the condition or increasing infection risk (Reyes-García et al., 2022; Hutchinson et al., 2024). Therefore, investigating the role of the neonatal gut microbiota in NRDS is essential.

Probiotics are live microorganisms that confer health benefits to the host. They are commonly used to maintain and restore the balance of gut microbiota (Sanders et al., 2011). Probiotics exert their effects by secreting metabolites such as short-chain fatty acids (e.g., butyrate) and lactate. These metabolites reduce gut pH, inhibit pathogen proliferation, and support a healthy microbiota structure (Ikeda et al., 2022). Recently, probiotics have been widely used in managing various diseases in neonates and PTIs (Samara et al., 2022). Probiotics have shown potential mechanisms for slowing disease progression, particularly in studies related to acute respiratory distress syndrome (ARDS). Studies suggest that *Lactobacillus rhamnosus* reduces inflammation in both the lungs and gut by secreting short-chain fatty acids and modulating neutrophil activity (Olimpio et al., 2022; Sapra et al., 2024). These findings suggest that probiotics, by modulating immune responses and altering microbiota composition, may offer therapeutic potential in managing respiratory diseases.

This study aimed to investigate the gut microbiota characteristics in NRDS infants and evaluate the effects of probiotic supplementation on microbial composition and recovery. Given that all participants received antibiotic treatment due to lung developmental immaturity or infections, we hypothesized that probiotic intervention could alleviate intestinal dysbiosis in NRDS infants due to antibiotics and promote microbial and disease recovery. To test this hypothesis, we compared gut microbiota composition before and after probiotic intervention and assessed microbial differences between infants receiving antibiotics alone and those receiving antibiotics with probiotics. The findings of this study will provide scientific evidence supporting the clinical use of probiotics as adjunct therapy for NRDS, offering new insights into managing neonatal gut health and rehabilitation strategies for respiratory distress syndrome.

## Materials and methods

### Protocol approvals and patient consent

The study was approved by the Ethics Committee of the First Affiliated Hospital of Zhengzhou University (2023-KY-0505-002, 2017-KY-12) and was conducted in accordance with the Declaration of Helsinki. Informed consent was obtained from all guardians prior to the collection of data and fecal samples.

### Participant information

This study enrolled 55 preterm infants (PTIs) diagnosed with NRDS and 26 PTIs without NRDS from the Neonatal Intensive Care Unit (NICU) of the First Affiliated Hospital of Zhengzhou University between March 2023 and June 2024. NRDS was diagnosed through clinical evaluation, arterial blood gas analysis, chest X-ray, ultrasound, and fluid culture. Exclusion criteria included respiratory dysfunction caused by congenital anomalies, gastrointestinal disorders (e.g., NEC, Hirschsprung disease, and intestinal atresia), missing clinical data, and withdrawal of participants at the request of their guardians. A total of 34 samples were collected from the NRDS group, and 26 were obtained from the non-NRDS group. Among the NRDS infants, one infant received neither antibiotics nor probiotics due to parental refusal of treatment and was included in the baseline analysis. The remaining 33 treated NRDS infants all exhibited lung developmental immaturity due to prematurity (with/without secondary infections) and received antibiotics therapeutically or prophylactically before sepsis was ruled out. Retrospective review indicated that probiotic administration followed clinical discretion. Participants were stratified into: (1) antibiotic-only (TA, *N* = 30) and (2) antibiotic plus probiotics (TB, *N* = 25). Samples were collected pre-and post-treatment (pre-TA = 20, post-TA = 30; pre-TB = 13, post-TB = 25) to assess probiotic efficacy. Fecal 16S rRNA sequencing data from healthy one-month-old children (Control-M1, *N* = 49), sourced from a prior study conducted by our team (with the corresponding author’s approval), were included to assess the post-treatment gut microbiota composition in neonates (Ma X. et al., 2023).

Perinatal clinical data, including Apgar scores, feeding methods, arterial blood gas analysis, and other blood results, were collected from all participants. The antibiotics used in this study were cephalosporins (ceftazidime or cefoperazone-sulbactam), and the probiotic was a two-strain formulation of *Clostridium butyricum* powder, containing *Clostridium butyricum* (≥1.0 × 10^7^ CFU/g) and *Bifidobacterium infantis* (≥1.0 × 10^6^ CFU/g). This probiotic combination has been widely used in neonatal clinical practice, and previous studies have demonstrated its safety in preterm infants, showing no significant risk of sepsis or colonization failure (Ren and Wang, 2010; Li et al., 2023).

### Sample collection

Fecal samples were collected within 48 h of birth and after recovery, and stored in sterile plastic tubes. The samples were then stored within 2 h at the Biobank of The First Affiliated Hospital of Zhengzhou University and the National Human Genetic Resources Sharing Service Platform (Grant No. 2005DKA21300) at −80°C until DNA extraction.

### DNA extraction

DNA was extracted from fecal samples using the E.Z.N.A.® Stool DNA Kit (Omega Bio-tek, Inc., GA) following the manufacturer’s protocol. Quantification was performed with a Qubit® 2.0 Fluorometer (Invitrogen, Carlsbad, CA, United States), while fragment size and integrity were assessed by 1.5% agarose gel electrophoresis. The extracted DNA was stored at −20°C until further analysis.

### PCR amplification and MiSeq sequencing

For the analysis of the V3 and V4 regions of the 16S ribosomal RNA (rRNA) gene, universal primers 341F (5′-CCTACGGGNGGC WGCAG-3′) and 805R (5′-GACTACHVGGGTATCTAATCC-3′) were used to amplify the specific regions. PCR reactions were conducted in an EasyCycler 96 PCR system (Analytik Jena Corp., AG) using the subsequent protocol: an initial denaturation of 3 min at 95°C, ensued by 21 cycles comprising a 0.5-min denaturation at 94°C, a 0.5-min annealing at 58°C, and a 0.5-min elongation at 72°C. The process concluded with a final extension at 72°C for 5 min. The products from different samples were mixed at equal ratios for sequencing according to the manufacturer’s instructions, and sequencing was performed on the Illumina MiSeq platform at Shanghai Mobio Biomedical Technology Co. Ltd.

### Data processing

Python scripts in the Quantitative Insights Into Microbial Ecology (QIIME 2) software pipeline were used to preprocess sequence data (Bolyen et al., 2019). After data preprocessing, the DADA2 algorithm was used to process the valid sequences from all samples and perform quality control. This involved removing duplicate sequences, denoising, and assembling the sequences. Subsequently, amplicon sequence variants (ASV) tables and sequences were generated, and chimeric sequences were removed (Callahan et al., 2016). The abundance of ASVs was normalized to the relative abundance of each sample based on the SILVA^2^ (release 138^1^) and NT-16S database (release 20230718).

### Bacterial diversity and taxonomic analysis

Alpha and beta diversity analyses were conducted to assess microbial diversity within and between samples. Bacterial community abundance was estimated using the Ace and Chao estimators. Venn diagrams were used to visualize the common and unique ASVs across different groups. Heatmap Builder was used to generate heatmaps for visualizing the dominant species across groups. Non-Metric Multidimensional Scaling (NMDS) and Principal Coordinates Analysis (PCoA) were conducted using the “Vegan” R package to explore microbial community differences between samples. Unweighted UniFrac distances were calculated with the “phyloseq” R package (McMurdie and Holmes, 2013). The Adonis and Anosim tests were used to assess the significance of inter-group differences, and ANOVA was applied to further analyze sample distribution differences between the two groups (Anderson, 2006). Evolutionary relationships between bacteria were visualized using a phylogenetic tree.

Bacterial taxonomy was analyzed at multiple taxonomic levels, including genus, family, order, class, and phylum. The Wilcoxon rank-sum test was used to assess the differences in microbiota between the two groups. Linear discriminant effect size (LEfSe) analysis was conducted to identify significant differences in species composition and community structure. Additionally, linear discriminant analysis (LDA) was used to evaluate the effect sizes of individual features, with a cut-off value of LDA score (log10) ≥ 3 (Segata et al., 2011). Pearson’s correlation coefficient was used to generate a heatmap, assessing the correlation between the microbiota and clinical data. The random forest model was employed to classify the samples. The area under the receiver operating characteristic (ROC) curve (area under the curve, AUC) was calculated to assess the diagnostic performance of the altered microbiota in predicting NRDS.

### Gene function prediction

PICRUSt2 (Phylogenetic Investigation of Communities by Reconstruction of Unobserved States) was used to predict the functional profile of the microbiota based on 16S rRNA gene sequences. This method established the abundance of Kyoto Encyclopedia of Genes and Genomes (KEGG) pathways/modules and KEGG orthology (KO). The 16S rRNA gene sequencing data were then matched with a database that includes established metabolic functions, aiming to predict the bacterial metabolic potential (Langille et al., 2013).

### Statistical analysis

Data were analyzed using R software (version 4.3.1) and SPSS software (version 25.0, SPSS Inc., United States). Normally distributed data are expressed as mean ± standard deviation and analyzed using independent t-tests or one-way analysis of variance (ANOVA). Non-normally distributed data are presented as median (interquartile range) and assessed using non-parametric tests, including the Mann–Whitney U test and Kruskal-Wallis test. Categorical data were analyzed using chi-square tests, continuity-corrected chi-square tests, or Fisher’s exact tests. The *post hoc* power analysis on the smallest sample group confirmed sufficient power (>92%) to detect significant differences in alpha diversity metrics (Ace and Chao). All statistical tests were two-tailed, and *p* < 0.05 was considered statistically significant. Significance levels were set at *p* < 0.05, denoted by “*,” *p* < 0.01 by “**,” and *p* < 0.001 by “***.”

## Results

### Clinical characteristics of the included subjects

**Figure 1:**
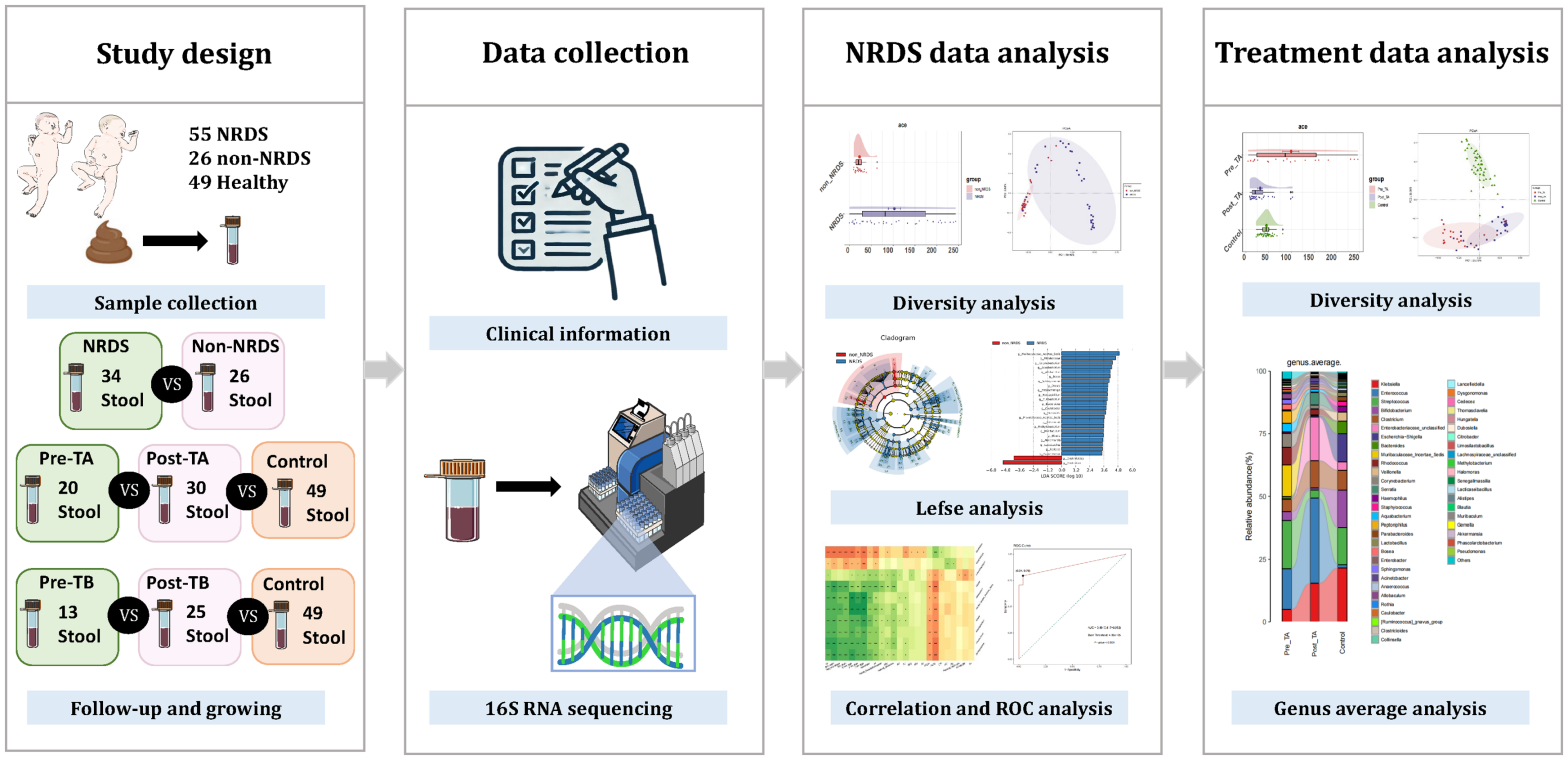
The study design is shown in the figure.

The research route of this study is shown in Figure 1. A total of 115 fecal samples were collected in this study, comprising 34 from the NRDS group, 26 from the non-NRDS group, 20 from the Pre-TA group, 30 from the Post-TA group, 13 from the Pre-TB group, and 25 from the Post-TB group. The Pre-TA and Pre-TB groups are included within the 34 samples of the NRDS group.

Table 1 summarizes the demographic and clinical characteristics of the patients included in this study. Compared with the non-NRDS group, the NRDS group exhibited significantly higher prenatal BMI, maternal hypertension rate, PaCO_2_, HCO3^−^, LYM counts, and AST levels (*p* < 0.05). In contrast, the NRDS group had significantly lower gestational age (GA), birth weight, birth length, and head circumference than the non-NRDS group (*p* < 0.05), indicating developmental impairment. For Apgar scores, the NRDS group had lower values at 1, 5, and 10 min relative to the non-NRDS group. With respect to feeding methods, the NRDS group exhibited a higher proportion of partial breastfeeding than the non-NRDS group. Blood test results indicated that WBC counts and NEU levels were lower in the NRDS group than in the non-NRDS group. No significant differences were observed in RBC, HGB, PLT, Urea, CREA, UA, ALT, TBIL, DBIL, or CRP levels. Clinical data for both groups are provided in Supplementary Table 1. Supplementary Table 2 presents the demographic and clinical profiles of the Pre-TA and Pre-TB groups. Only pregnancy weight gain differed between the Pre-TA and Pre-TB groups, no significant differences were observed for other clinical characteristics, Apgar scores, feeding patterns, blood gas parameters, or blood test results.

**Table 1:** Characteristics of the NRDS and non-NRDS groups.

	NRDS (*N* = 34)	Non-NRDS (*N* = 26)	*P*-value
Prenatal BMI, kg/m^2^	30.36 ± 4.02	27.56 ± 3.46	0.006965
Pregnancy weight gain, kg	12.08 ± 5.15	11.77 ± 4.18	0.805987
Gender, female	14 (41.2%)	10 (38.5%)	0.831547
Gestational age (GA), weeks	31.5 (29.0–34.0)	35.5 (34.0–36.0)	8.09E-09
Birth weight, g	1755.00 ± 649.55	2624.23 ± 529.05	7.28E-07
Birth length, cm	42.00 (39.50–45.25)	47.00 (46.00–48.25)	3.77E-07
Head circumference, cm	29.75 (21.00–32.00)	33.25 (32.00–35.00)	4.97E-07
Cesarean section (CS)	30 (88.2%)	25 (96.2%)	3.95E-01
*In vitro* fertilization (IVF)	4 (11.8%)	5 (19.2%)	1.92E-01
Maternal hypertension	11 (32.4%)	1 (3.8%)	0.015959
Maternal diabetes	9 (26.5%)	6 (23.1%)	0.763545
**Apgar score**			
1 min-Apgar	8 (7–9)	10 (9–10)	0.000001
5 min-Apgar	9 (9–10)	10 (10–10)	0.000049
10 min-Apgar	9.5 (9–10)	10 (10–10)	0.000074
Resuscitation, yes	8 (23.5%)	0 (0%)	0.007707
**Nutrition**			
Partially breastfed	18 (52.9%)	3 (11.5%)	0.002222
Nonbreastfed	16 (47.1%)	23 (88.5%)	
**Arterial blood gas analysis**			
pH	7.28 ± 0.74	7.39 ± 0.63	4.21E-08
Partial pressure of oxygen (PaO_2_), mmHg	65.00 (55.75–70.00)	72.50 (53.50–80.00)	0.514602
Partial pressure of carbon dioxide (PaCO_2_), mmHg	46.0 (39.0–54.5)	34.5 (30.0–37.0)	4.65E-07
Bicarbonate radical (HCO3^−^), mmol/L	23.23 ± 3.23	21.37 ± 2.40	0.013065
Buffer excess (BE), mmol/L	−5.26 ± 2.63	−3.53 ± 1.85	0.004139
Lactate (Lac), mmol/L	1.40 (1.10–2.15)	1.50 (1.38–2.53)	0.894075
Anion gap (AG)	12.00 (9.75–14.00)	14.00 (12.75–15.25)	0.057169
**Blood tests**			
Red blood cell (RBC), ×10^12^/L	4.62 (4.26–4.84)	4.41 (4.05–4.82)	0.239442
Hemoglobin (HGB), g/L	170.00 (152.75–182.25)	163.50 (151.50–177.00)	0.241625
White blood cell (WBC) count, ×10^9^/L	9.33 ± 4.02	11.76 ± 3.75	0.020477
Neutrophil (NEU), %	43.24 ± 16.41	53.91 ± 14.04	0.01024
Lymphocyte (LYM), %	47.10 ± 15.67	34.68 ± 13.38	0.00201
Platelet (PLT), ×10^9^/L	248.72 ± 45.57	263.19 ± 62.83	0.30181
Urea, mmol/L	3.89 (3.11–4.72)	3.25 (2.76–3.94)	0.293692
Creatinine (CREA), μmol/L	56.00 (47.75–70.25)	51.00 (45.75–57.75)	0.249976
Uric acid (UA), μmol/L	332.50 (263.25–474.50)	316.00 (291.75–376.25)	0.600116
Alanine aminotransferase (ALT), U/L	4.00 (2.00–7.00)	4.00 (2.75–5.25)	0.929557
Aspartate aminotransferase (AST), U/L	37.50 (28.00–50.75)	27.00 (25.00–34.25)	0.002131
Total Bilirubin (TBIL), μmmol/L	39.10 (32.83–50.65)	36.40 (31.08–42.45)	0.067334
Direct Bilirubin (DBIL), μmmol/L	6.50 (4.65–8.35)	6.30 (5.40–11.08)	0.132808
C-reactive protein (CRP) level, mg/L	0.38 (0.14–0.54)	0.19 (0.12–0.50)	0.10278

### Increased gut microbiota diversity in NRDS

**Figure 2:**
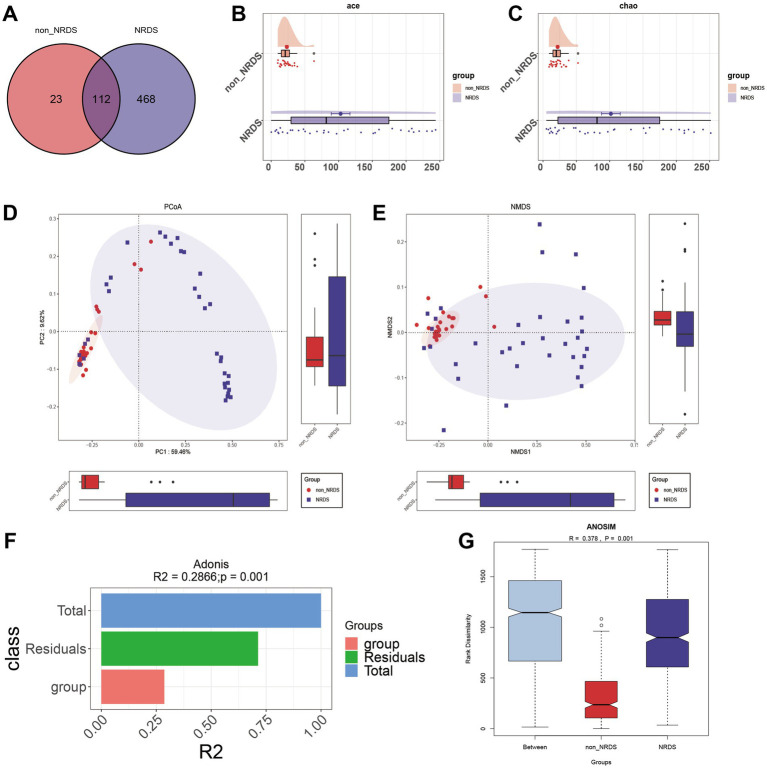
Comparison of alpha and beta diversity between the NRDS and non-NRDS groups. **(A)** The Venn diagram demonstrates the ASVs shared between the two groups. **(B)** Alpha diversity based on the ACE index between the groups. **(C)** Alpha diversity based on the Chao index between the groups. **(D)** PCoA based on the unweighted UniFrac distance. **(E)** NMDS analysis. **(F,G)** Adonis and ANOSIM analyses indicate significant differences between the groups. NRDS, neonatal respiratory distress syndrome; ASV, amplicon sequence variants; NMDS, non-metric multidimensional scaling; PCoA, principal coordinate analysis.

Venn analysis revealed that among the 603 ASVs, 112 were shared by both NRDS and non-NRDS groups, with 468 unique to NRDS and 23 unique to non-NRDS (Figure 2A; Supplementary Table 3). Compared with the non-NRDS group, ACE and Chao estimators indicated that the NRDS group had significantly higher fecal microbial diversity (*p* < 0.001, Figures 2B,C). To assess similarities between microbial communities, PCoA and NMDS analyses were performed using unweighted UniFrac distances (Figures 2D,E). Both methods highlighted significant differences in fecal microbial structures between the NRDS and non-NRDS groups. The Adonis analysis indicated significant intergroup differences (Figure 2F, R2 = 0.2866, *p* = 0.001). ANOSIM analysis of intergroup distances revealed that the two subgroups were clearly distinguishable (Figures 2G, *R* = 0.378, p = 0.001).

### Characteristic community structure of the gut microbiota in NRDS

**Figure 3:**
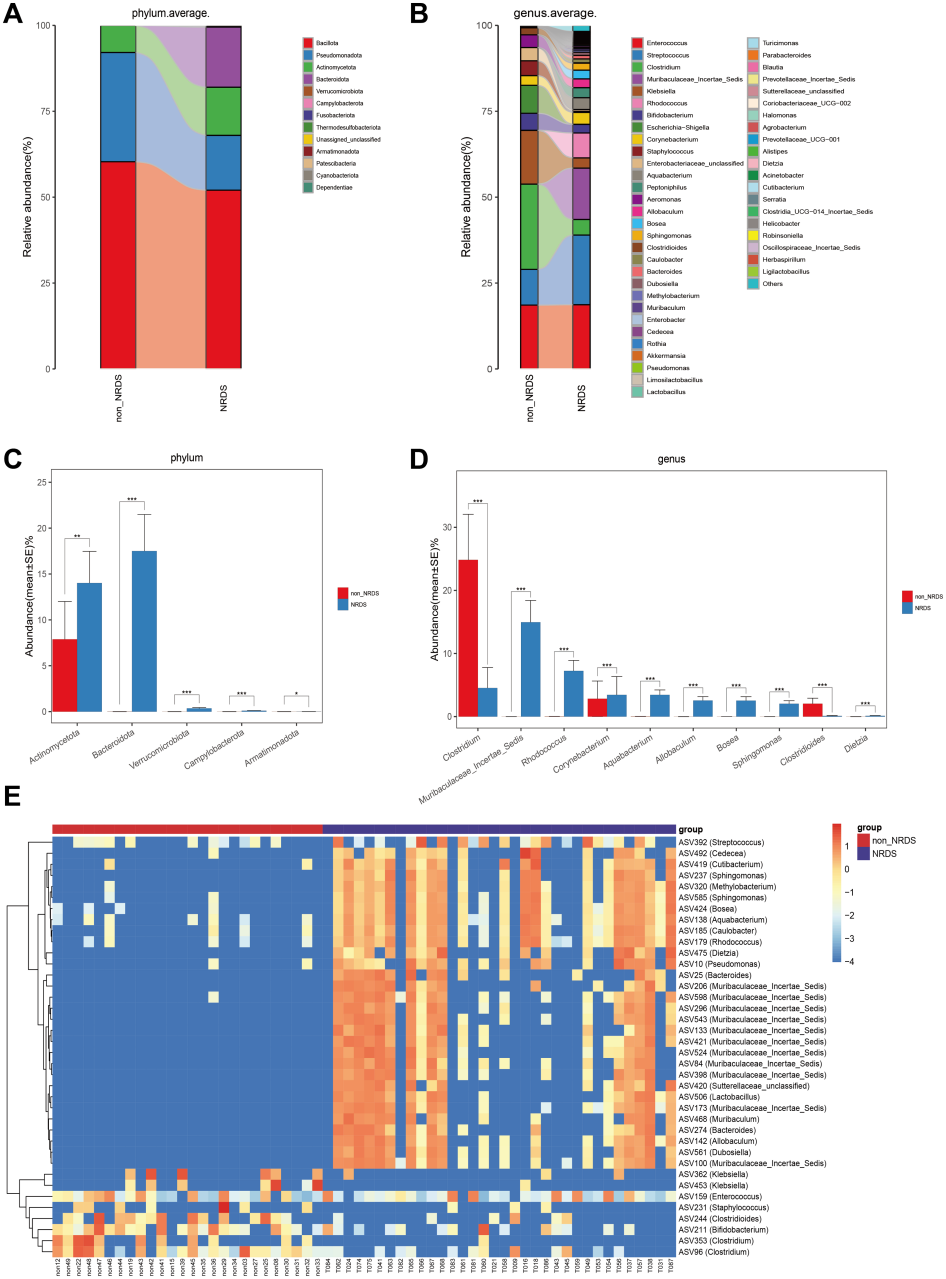
Composition and comparison of fecal microbiota between the NRDS and non-NRDS groups. **(A)** Phylum-level composition of fecal microbiota in both groups. **(B)** Genus-level composition of fecal microbiota in both groups. **(C,D)** The relative abundance differences of key bacteria at the phylum and genus levels. The relative abundance of each bacteria was represented by the mean ± SE. Significance of the differences in relative abundance was evaluated using Wilcoxon rank-sum tests (*p < 0.05; **p < 0.01; and ***p < 0.001). **(E)** The heatmap illustrates significantly different ASVs and bacterial communities between the two groups, where red indicates high abundance and blue indicates low abundance. Each row represents an ASV. NRDS, neonatal respiratory distress syndrome; ASV, amplicon sequence variants.

Further analyses were conducted to examine the gut microbiota composition in the NRDS and non-NRDS groups, including alterations at the phylum and genus levels. The average phylum-and genus-level compositions of the microbial communities in both groups are displayed in Figures 3A,B. Phylum-and genus-level fecal bacterial compositions for each individual sample are presented in Supplementary Figures 1A,B. At the phylum level, Bacillota, Pseudomonadota, Actinomycetota, and Bacteroidota together accounted for 99% of the total average relative abundance, constituting the four dominant phyla in both groups. At the genus level, *Enterococcus*, *Streptococcus*, *Clostridium*, *Muribaculaceae Incertae Sedis*, *Klebsiella*, *Rhodococcus*, and *Bifidobacterium* collectively exceeded 70% of the total average relative abundance, thereby forming the seven dominant genera in both groups. The Wilcoxon rank-sum test was then employed to identify significant differences in microbial composition between the NRDS and non-NRDS groups. At the phylum level, Actinomycetota and Bacteroidota exhibited significantly higher abundances in the NRDS group than in the non-NRDS group (Figure 3C). At the genus level, *Clostridium* and *Clostridioides* displayed significantly greater abundances in the non-NRDS group than in the NRDS group. In contrast, *Muribaculaceae Incertae Sedis*, *Rhodococcus*, *Corynebacterium*, *Aquabacterium*, *Allobaculum*, *Bosea*, *Sphingomonas*, and *Dietzia* were present at significantly higher abundances in the NRDS group than in the non-NRDS group (Figure 3D). A heatmap illustrates the microbial community differences between the two groups (Figure 3E).

**Figure 4:**
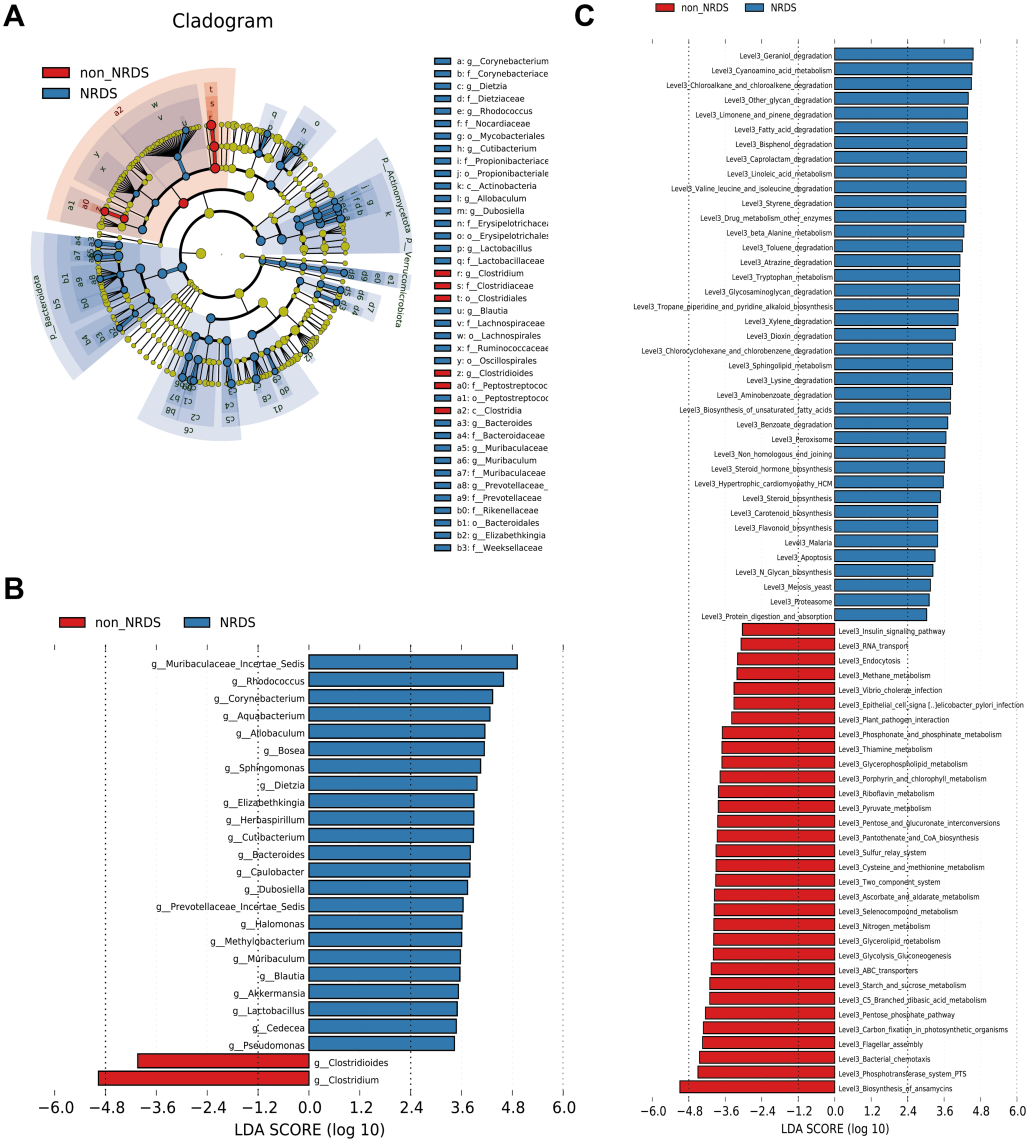
LEfSe analysis identified the characteristic microbial community composition and metabolic function predictions for NRDS. **(A)** The cladogram constructed with LEfSe indicates the significantly different taxa between NRDS and non-NRDS groups. **(B)** The bar chart shows significant differences in gut microbiota between the two groups. **(C)** The bar chart presents the predictive functional analysis of the metagenome using the LEfSe method, identifying significantly enriched KEGG pathways between the two groups. Higher LDA scores indicate greater importance of microbial biomarkers and KEGG pathways. An LDA score exceeding 3 and a *p*-value below 0.05 are considered indicative of significant differences. LEfSe, linear discriminant analysis effect size; NRDS, neonatal respiratory distress syndrome; LDA, linear discriminant analysis; KEGG, Kyoto Encyclopedia of Genes and Genomes.

LEfSe was employed to identify specific bacterial taxa associated with NRDS. The cladogram illustrating fecal microbial structure and major bacterial populations revealed the most pronounced taxonomic differences between the NRDS and non-NRDS groups (Figure 4A). Similarly, the cladogram of fecal microbial communities in NRDS and non-NRDS indicated substantial differences (Figure 4B). The top ten taxa identified by LDA scores are listed in Supplementary Table 4, suggesting that gut microbiota alterations occur in NRDS. Predictions of microbial metabolic functions using PICRUSt2 evaluated potential microbial roles associated with NRDS. These results again demonstrated notable distinctions between the two groups (Figure 4C). In the NRDS group, the top ten pathways identified by LDA scores included linoleic acid metabolism, fatty acid degradation, and valine, leucine, and isoleucine degradation. These pathways were associated with PS synthesis, cellular energy metabolism, and repair functions, contributing to metabolic imbalance and pulmonary repair in NRDS. In the non-NRDS group, the top ten LDA-scored pathways included ABC transporters, the phosphotransferase system (PTS), and glycolysis/gluconeogenesis. These pathways were closely linked to pulmonary gas exchange, metabolic processes, and material transport.

### Correlations between microbiota and clinical characteristics

**Figure 5:**
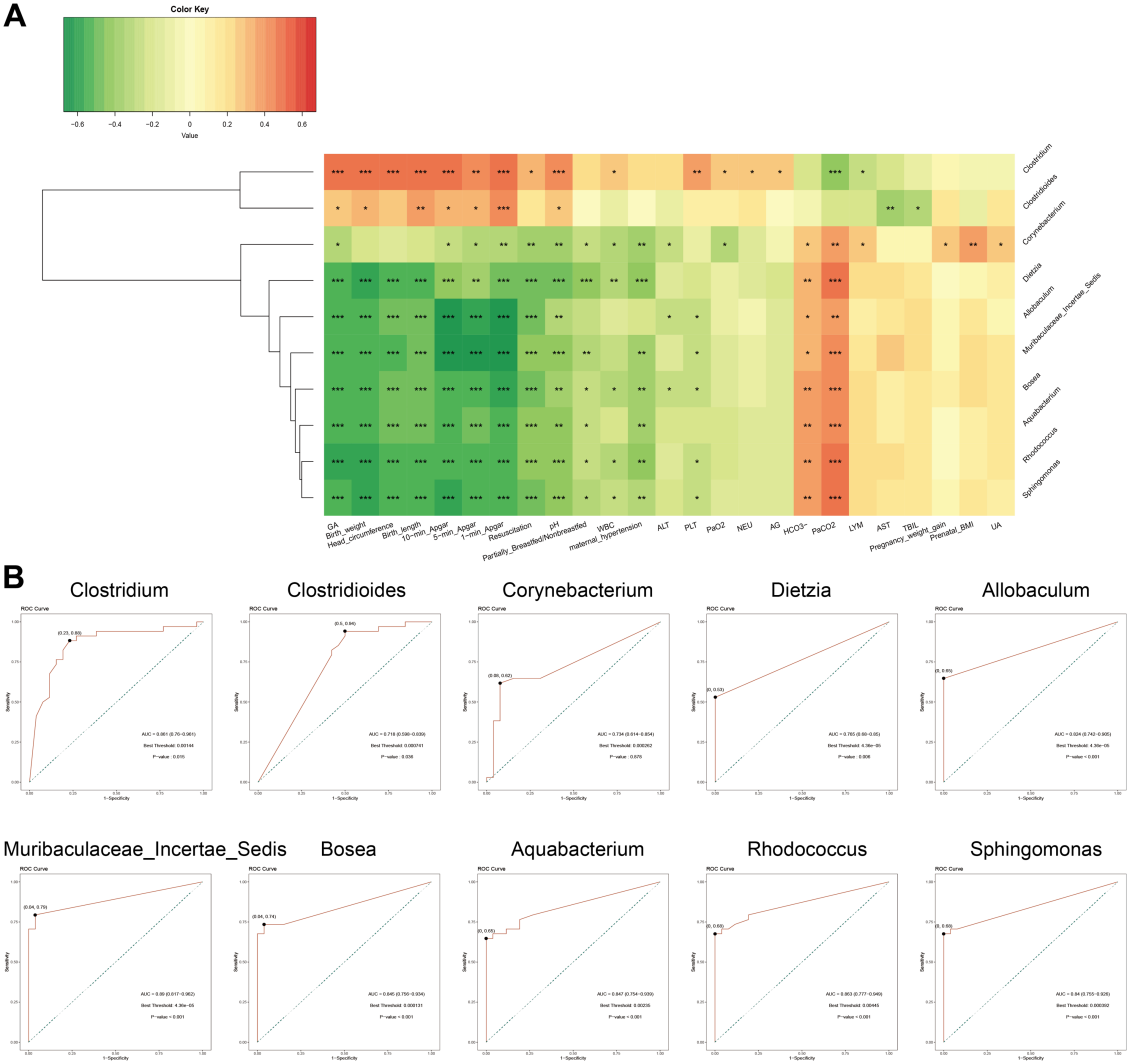
Correlation and ROC analyses of signature microbiota in NRDS. **(A)** Correlation analysis between the top 10 signature microbiota in NRDS and clinical data (*p < 0.05; **p < 0.01; and ***p < 0.001; red denotes positive correlation, green denotes negative correlation). **(B)** ROC analysis of the top 10 signature microbiota in NRDS (the X-axis represents 1-specificity, and the Y-axis indicates sensitivity. Marked points on the curves signify optimal thresholds). NRDS, neonatal respiratory distress syndrome; ROC, receiver operating characteristic; AUC, area under the curve.

To investigate the associations between NRDS-characteristic microbiota and clinical characteristics, we selected the top ten taxa identified by LEfSe analysis (based on LDA scores): *Clostridium*, *Muribaculaceae Incertae Sedis*, *Rhodococcus*, *Corynebacterium*, *Aquabacterium*, *Allobaculum*, *Bosea*, *Sphingomonas*, *Clostridioides*, and *Dietzia* (Figure 4B). Among these, *Clostridium* and *Clostridioides* were highly abundant in the non-NRDS group, whereas the other eight taxa showed higher abundance in the NRDS group. Spearman correlation analysis indicated that GA, birth weight, head circumference, birth length, Apgar score, pH, feeding method, WBC, maternal hypertension, ALT, PLT, PaO_2_, NEU, and AG were significantly positively correlated with *Clostridium* and *Clostridioides*, but significantly negatively correlated with the other eight taxa. HCO3^−^ and PaCO_2_ exhibited significant negative correlations with *Clostridium* and *Clostridioides*, but significant positive correlations with the other eight taxa (Figure 5A; Supplementary Table 5). ROC analyses were conducted to evaluate the diagnostic value of these ten characteristic microbial taxa in NRDS. The results demonstrated that all AUC values exceeded 0.7, indicating that these ten taxa possess strong diagnostic accuracy for NRDS (Figure 5B; Supplementary Table 6).

### Probiotics remodel the disturbed intestinal microbiota diversity in NRDS

**Figure 6:**
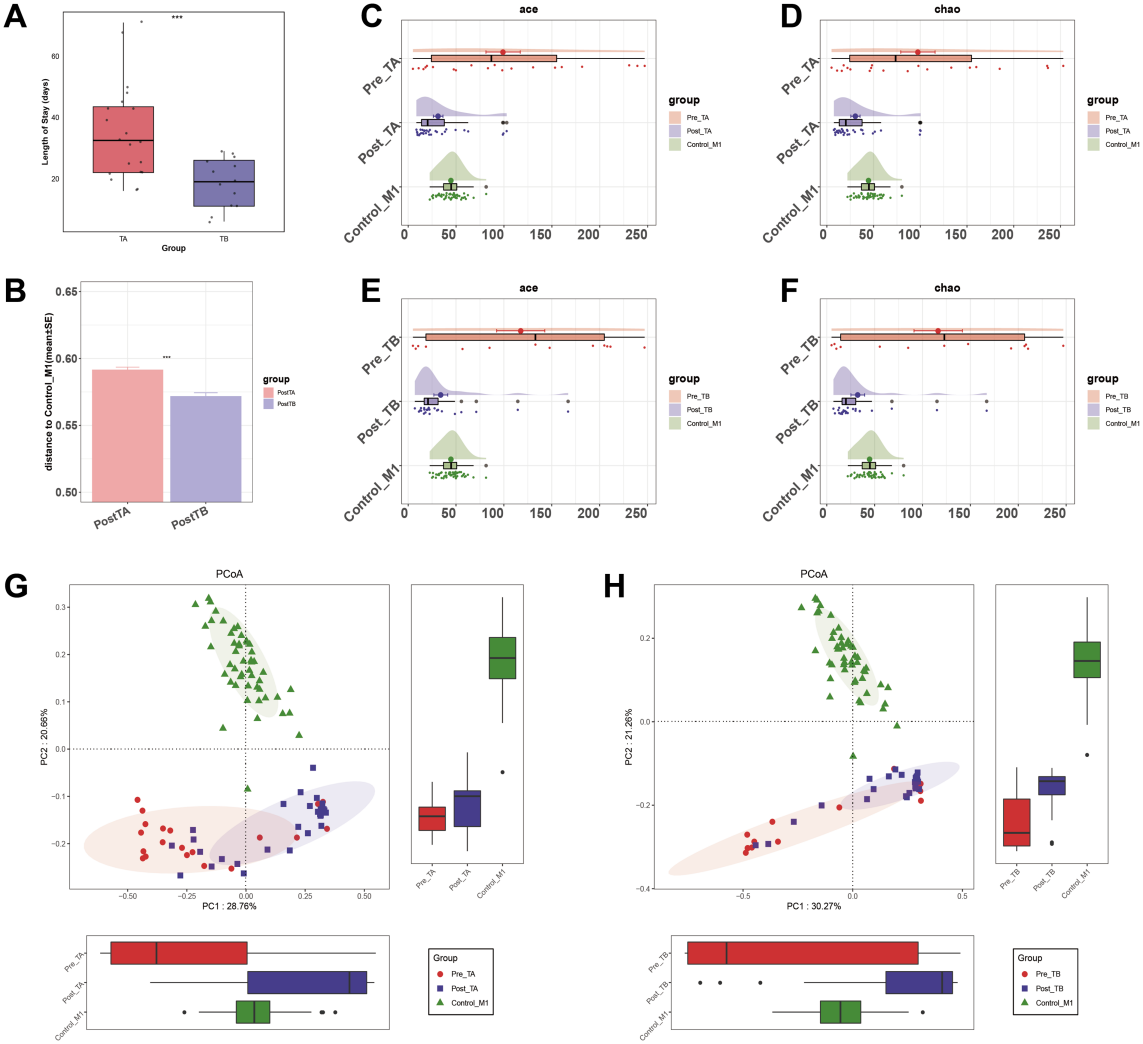
Comparison of alpha diversity and beta diversity under different treatment methods. **(A)** Comparison of hospitalization lengths between the two treatment methods. **(B)** Comparison of differences between the two treatment methods post-treatment and Control-M1 based on unweighted UniFrac distance (*p < 0.05; **p < 0.01; and ***p < 0.001). **(C,D)** Alpha diversity, based on the ACE and Chao indices, among Pre-TA, Post-TA, and Control-M1 groups. **(E,F)** Alpha diversity, based on the ACE and Chao indices, among Pre-TB, Post-TB, and Control-M1 groups. **(G,H)** PCoA based on the unweighted UniFrac distance for different treatment methods. Pre, pre-treatment; Post, post-treatment; TA, antibiotic-only treatment group; TB, antibiotic plus probiotics treatment group; M1, one month; PCoA, principal coordinate analysis.

The TB group had a significantly shorter hospital stay than the TA group (*p* < 0.001, Figure 6A). To explore the role of the gut microbiome in the progression of NRDS, we compared the intestinal microbial differences between treated patients from both groups and healthy one-month-old infants. The unweighted UniFrac distance between Post-TB and Control-M1 was significantly shorter than that observed for Post-TA (*p* < 0.001, Figure 6B). Compared to Pre-TA, the fecal microbial diversity assessed by ACE and Chao estimators was significantly decreased in Post-TA and Control-M1 (*p* < 0.001, Figures 6C,D). In comparison to Pre-TB, fecal microbial diversity was significantly decreased in Post-TB and Control-M1 as well (*p* < 0.001, Figures 6E,F). Based on unweighted UniFrac distances, PCoA analysis was performed (Figures 6G,H). The distance between Post-TB and Control-M1 was significantly shorter than that observed between Post-TA and Control-M1. These findings suggest that probiotics can remodel intestinal microbiota diversity in NRDS infants, potentially reducing disease duration. Therefore, we further investigated which dysbiotic gut microbial communities were restored following probiotic treatment.

### Probiotics restore the altered gut bacterial taxa in NRDS

**Figure 7:**
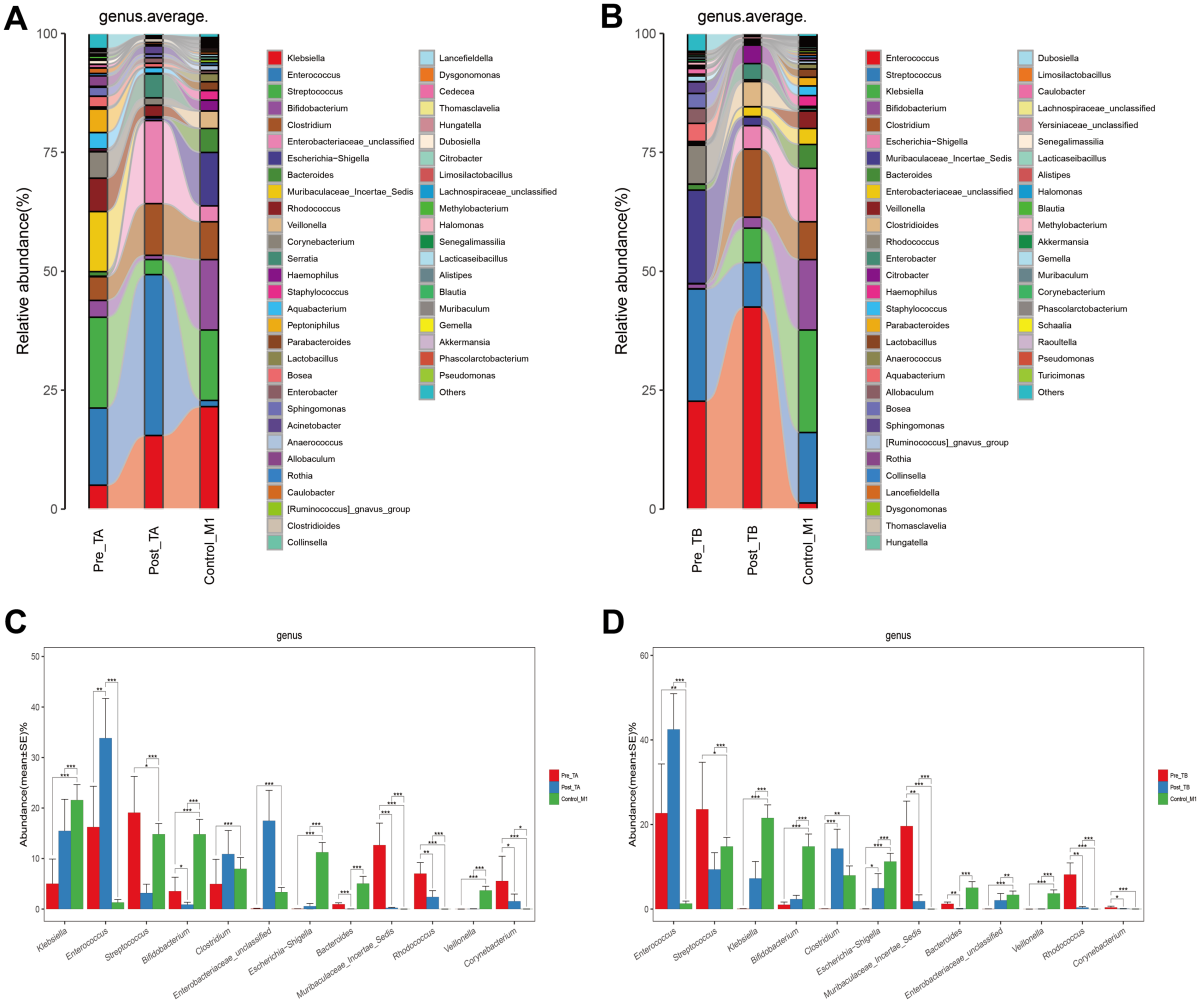
Composition and comparison of the fecal microbiota under different treatment methods. **(A)** Composition of fecal microbiota at the genus level among Pre-TA, Post-TA, and Control-M1 groups. **(B)** Composition of fecal microbiota at the genus level among Pre-TB, Post-TB, and Control-M1 groups. **(C,D)** Relative abundance differences of key microbiota at the genus level in the TA and TB treatment protocols. The relative abundance of each bacteria was represented by the mean ± SE. Significance of the differences in relative abundance was evaluated using Wilcoxon rank-sum tests (*p < 0.05; **p < 0.01; and ***p < 0.001). Pre, pre-treatment; Post, post-treatment; TA, antibiotic-only treatment group; TB, antibiotic plus probiotics treatment group; M1, one month.

To investigate how probiotics regulate gut dysbiosis in NRDS infants, we further analyzed the gut microbial composition and genus-level alterations after applying two treatment methods. The mean genus-level compositions for both groups are displayed in Figures 7A,B, and individual sample compositions are provided in Supplementary Figures 2A,B. At the genus level, *Klebsiella*, *Enterococcus*, *Streptococcus*, *Bifidobacterium*, *Clostridium*, *Enterobacteriaceae unclassified*, *Escherichia-Shigella*, *Bacteroides*, *Muribaculaceae Incertae Sedis*, *Rhodococcus*, *Veillonella*, and *Corynebacterium* collectively constituted over 75% of the total relative abundance. These 12 genera represented the predominant taxa in both treatment groups and in normal one-month-old infants. The Wilcoxon rank-sum test showed that under the TB treatment, the abundances of *Streptococcus*, *Bifidobacterium*, *Clostridium*, *Enterobacteriaceae unclassified*, and *Escherichia-Shigella* more closely resembled those observed in normal one-month-old infants than did those under the TA treatment (Figures 7C,D). Box plots indicated that the relative abundances of *Streptococcus*, *Bifidobacterium*, and *Clostridium* more closely matched those seen in normal one-month-old infants (Supplementary Figures 3A–C). Taken together, these findings indicate that adding probiotics helps restore the gut microbial imbalance altered by NRDS, thereby promoting the recovery of affected infants.

## Discussion

This study investigated the gut microbiota characteristics of NRDS and innovatively conducted the first comprehensive assessment of the potential role of probiotics in regulating the gut microbiota and promoting disease recovery. By comparing the gut microbiota of NRDS and non-NRDS infants, significant differences in microbial diversity and composition between the two groups were revealed. Notably, the majority of NRDS cases in our study were mild, with only two cases having an oxygenation index (OI) > 8, while the remaining 32 cases were classified as mild NRDS. We found that the gut microbial diversity in NRDS infants was significantly higher than that in non-NRDS infants, a result previously reported by Bi et al. (2024). The study found that dysbiosis, particularly intestinal dysbiosis, has been associated with immune modulation, including differentiating T regulatory cells (Tregs) and T helper 17 (Th17) cells, which play key roles in pulmonary inflammatory conditions (Ziaka and Exadaktylos, 2024b). Therefore, changes in gut microbial diversity in NRDS may reflect early activation of the infants’ immune systems and the impact of incomplete lung development on the intestinal microbial communities (Yang et al., 2021; Zhou and Liao, 2021). Similar changes in microbial diversity have been reported in studies of other pulmonary and immune-related diseases, including COVID-19 and Kawasaki disease in children (Mańkowska-Wierzbicka et al., 2023; Teramoto et al., 2023).

At the phylum level, Actinomycetota and Bacteroidota abundances were significantly elevated in the NRDS group compared to the non-NRDS group. In Yang et al.’s study, neonatal ARDS exhibited higher abundances of Proteobacteria and Bacteroidota and lower abundances of Firmicutes in lower respiratory tract secretions. In the latest SILVA2 and NT-16S databases, Actinomycetota corresponds to Proteobacteria, and Bacillata corresponds to Firmicutes. This indicates similar intestinal microbial alterations in the gut and lower respiratory tract secretions of NRDS patients (Yang et al., 2024). At the genus level, pathogenic genera such as *Muribaculaceae Incertae Sedis*, *Rhodococcus* (Lin et al., 2019), and *Corynebacterium* (Burkovski, 2015) had higher abundances, whereas *Clostridium* was predominant in the non-NRDS group. Mendelian randomization analysis also revealed a significant positive correlation between certain genera, such as *Victivallis*, and IL-16. This correlation affects human immune and inflammatory responses and influences the occurrence and progression of ARDS (Ma J. et al., 2023). Alterations in the gut microbiota may affect pulmonary inflammatory responses and oxygen exchange functions, thereby exacerbating the clinical symptoms of NRDS.

Employing the gut microbiota as a novel biomarker for disease diagnosis, as well as for evaluating prognosis and recurrence, has been explored in numerous diseases. For instance, Zheng et al. integrated *Veillonella* and *Streptococcus pneumoniae* with TNM staging and AST to develop a new biomarker for predicting the early recurrence of HBV-related stem cell carcinoma (Zheng et al., 2023). In additional, Kartal et al. utilized a 27-taxon microbial model to diagnose pancreatic ductal adenocarcinoma with remarkably high accuracy (Kartal et al., 2022). In this study, ROC analysis assessed the diagnostic potential of the top ten LDA score ranked taxa for NRDS diagnosis. These taxa demonstrated strong discriminatory capability between NRDS and non-NRDS infants, all with AUC values above 0.7. This suggests that shifts in the gut microbiota serve as potential biomarkers. Notably, *Muribaculaceae Incertae Sedis* and *Allobaculum* exhibited abundance changes closely associated with Apgar score, pH, PaO_2_, and PaCO_2_ in NRDS infants. This may provide potential insights for future NRDS diagnosis and severity grading based on gut microbiota.

The gut-lung axis, as an important mechanism by which the gut microbiota regulates lung health and disease, plays a significant regulatory role in ARDS (Ziaka and Exadaktylos, 2024a,b). Regulation of the gut microbiota can influence the occurrence and progression of ARDS by triggering inflammatory responses, immune cell dysfunction, oxidative stress, apoptosis, autophagy, pyroptosis, and ferroptosis mechanisms (Jin et al., 2019; Li et al., 2021; Xu et al., 2022; Zhang et al., 2024). However, studies involving neonates remain relatively limited. In clinical treatments, NRDS infants, before sepsis is excluded, often receive therapeutic or prophylactic antibiotic therapy due to incomplete lung development and concurrent pulmonary infections, which significantly impacts the gut microbiota dysbiosis in NRDS infants (Sweet et al., 2023). The gut-lung axis plays a critical role in immune homeostasis and pulmonary health. Emerging evidence suggests that gut microbiota can regulate lung immune responses through the production of short-chain fatty acids (SCFAs), particularly butyrate, which has been shown to enhance Tregs differentiation and suppress pro-inflammatory cytokines (Hu et al., 2022; Huang et al., 2023; Lin et al., 2024). Additionally, gut microbiota alterations can influence intestinal permeability, allowing microbial metabolites to enter circulation, thereby modulating systemic inflammation and lung immune responses (Qin et al., 2022; Wei et al., 2024). The restoration of beneficial bacterial taxa observed in our study may have contributed to strengthening intestinal barrier integrity, thereby reducing systemic inflammation and supporting pulmonary recovery in NRDS infants. Additionally, probiotics, effective regulators of the gut microbiota, can mitigate antibiotic-induced dysbiosis by increasing the relative abundance of beneficial taxa and limiting the proliferation of opportunistic pathogens (Merenstein et al., 2021; Uttarwar et al., 2024).

Therefore, we further investigated the potential of probiotics in restoring gut microbiota dysbiosis in NRDS. Our research demonstrated that probiotic intervention significantly repaired the gut microbiota structure in NRDS infants, particularly by increasing the abundance of the genera *Streptococcus*, *Bifidobacterium*, and *Clostridium*. These three bacterial genera have been extensively studied in other diseases. For instance, within the *Streptococcus* genus, *Streptococcus thermophilus* is widely studied as a probiotic (Roux et al., 2022). *Bifidobacterium* has been validated for its critical role in preventing virus-induced pulmonary inflammation and injury (Khailova et al., 2014; Groeger et al., 2020). *Clostridium butyricum* has been identified as having potential value in enhancing the efficacy of lung cancer therapies (Tomita et al., 2022). Regarding other respiratory diseases, studies have found that adding probiotics can alleviate asthma symptoms and reduce pulmonary inflammation (Liu et al., 2021; Wenger et al., 2022). Our further findings demonstrated that oral probiotics promoted the restoration of specific beneficial bacterial taxa in NRDS infants, thereby reducing microbial composition discrepancies compared to healthy children and potentially contributing to disease recovery. This regulation of dysbiosis consequently exerts a positive impact on pulmonary inflammation and disease recovery in NRDS through the gut-lung axis. This offers potential therapeutic value for the clinical management of NRDS and the improvement of patient prognosis.

We acknowledge the limitations of this study. First, the limited sample size and the difficulty in collecting neonatal samples, due to enema and urine contamination, led to the exclusion of certain samples from this study. Second, given that the gut microbiota in neonates remains unstable during the first year after birth (Bäckhed et al., 2015), even though we excluded some severely deviated data, deviations in a small subset of samples could significantly impact the results. Third, group allocation was not randomized, as probiotics were administered at the discretion of the attending neonatologists. Although we controlled for baseline differences through statistical analyses, potential selection bias and unmeasured confounders may still exist. Moreover, although the Control-M1 group served as a baseline for healthy infants, feeding patterns were not matched. Given the limited sample size and challenges in neonatal sample collection, further stratification would have reduced available samples, hindering meaningful comparisons. This remains a potential confounding factor. Thus, future prospective randomized controlled trials (RCTs) will help verify our findings and address the limitations of the current study design. In addition, exploring the impact of probiotic intervention on the long-term prognosis of NRDS patients, especially regarding immune function restoration, intestinal barrier integrity, and pulmonary repair, possesses important clinical significance. Through such research, probiotics may serve as a safe and effective therapeutic approach, potentially offering new clinical strategies for the management and recovery of NRDS.

## Conclusion

This study found an increase in gut microbiota diversity in the NRDS group and observed changes in specific bacterial communities. These changes are closely related to clinical data of NRDS, such as Apgar score, pH, PaO_2_, and PaCO_2_, and can serve as novel biomarkers for NRDS. The addition of probiotic treatment regulated gut microbiota dysbiosis and reduced microbiota differences compared to normal age-matched children. This intervention improved the disease course in NRDS patients through the gut-lung axis, providing new insights for NRDS treatment.

## Data Availability

The original contributions presented in the study are publicly available. This data can be found here: https://www.ncbi.nlm.nih.gov/, accession numbers PRJNA1196624 and PRJNA665920.

## Footnotes

^1^ https://www.arb-silva.de/documentation/release138/

## Supplementary material

Supplementary Figure 1 Composition of fecal microbiota in each sample of the NRDS and non-NRDS groups. **(A)** Phylum-level composition of fecal microbiota in each sample of both groups. **(B)** Genus-level composition of fecal microbiota in each sample of both groups. NRDS, neonatal respiratory distress syndrome.

Supplementary Figure 2 Composition of the fecal microbiota in each sample under different treatment methods. **(A)** Composition of fecal microbiota at the genus level among Pre-TA, Post-TA, and Control-M1 groups. **(B)** Composition of fecal microbiota at the genus level among Pre-TB, Post-TB, and Control-M1 groups. Pre, pre-treatment; Post, post-treatment; TA, antibiotic-only treatment group; TB, antibiotic plus probiotics treatment group; M1, one month.

Supplementary Figure 3 Changes in relative abundance of three microbial communities under different treatment modalities. Differences in the relative abundance changes of *Streptococcus* **(A)**, *Bifidobacterium* **(B)**, and *Clostridium* **(C)** in TA and TB treatment modalities. The relative abundance of each bacteria was represented by the mean ± SE. Significance of the differences in relative abundance was evaluated using Wilcoxon rank-sum tests (**p* < 0.05; ***p* < 0.01; and ****p <* 0.001). Pre, pre-treatment; Post, post-treatment; TA, antibiotic-only treatment group; TB, antibiotic plus probiotics treatment group; M1, one month.

Supplementary Table 1 Clinical data of the NRDS and non-NRDS groups.

Supplementary Table 2 Characteristics of the pre-TA and pre-TB groups.

Supplementary Table 3 A total of 603 ASVs were identified.

Supplementary Table 4 Top ten genera based on LDA scores in LEfSe analysis.

Supplementary Table 5 Spearman correlation analysis between the top ten genera and clinical data.

Supplementary Table 6 AUC values and thresholds for the top ten genera.
