# Supplementary figures and images for: Gut microbiota characteristics in neonatal respiratory distress syndrome and the therapeutic potential of probiotics in recovery
Source: Front Microbiol. 2025 Apr 4;16:1544055. doi: 10.3389/fmicb.2025.1544055 (PMC12006762; doi:10.3389/fmicb.2025.1544055)

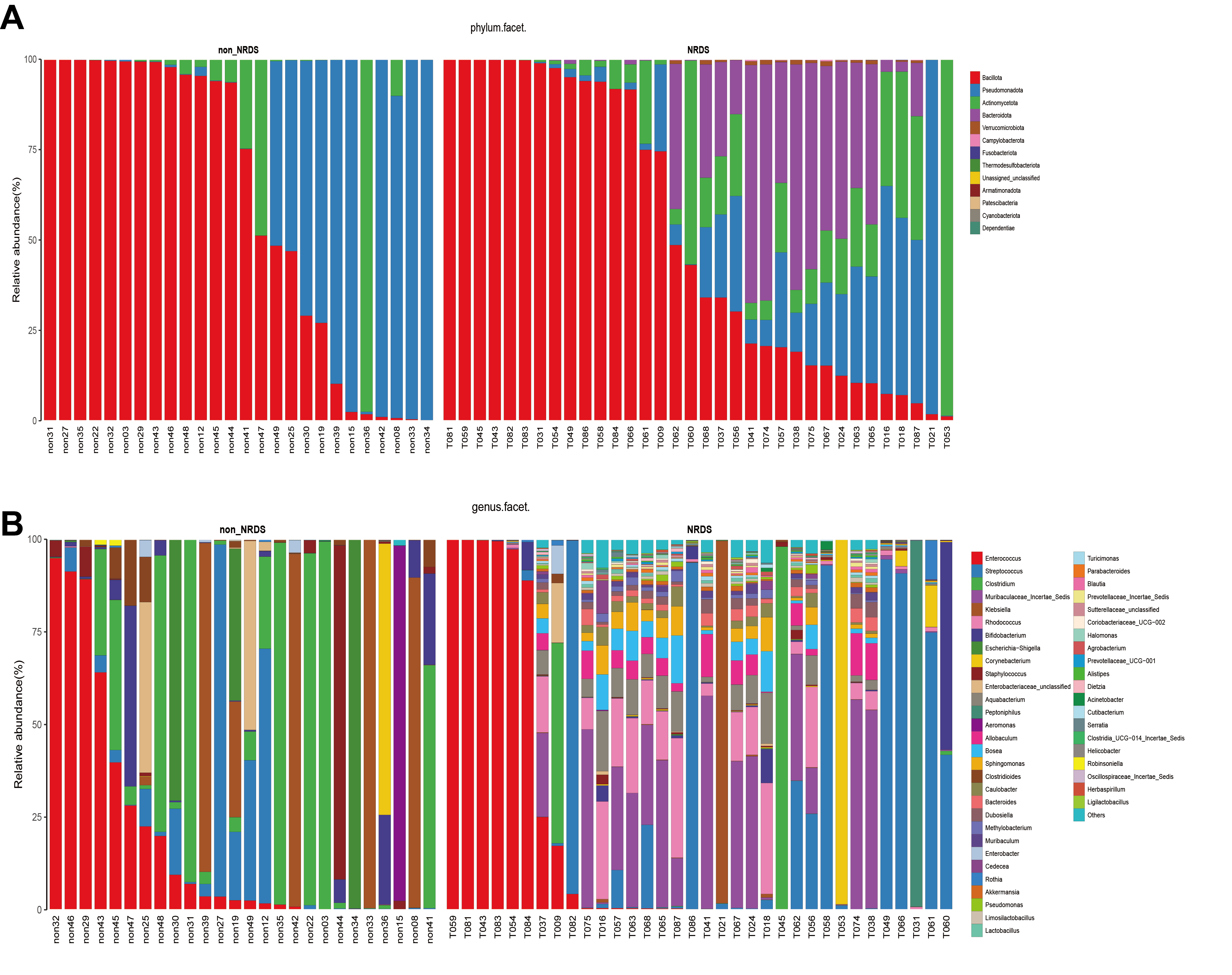

Supplement: Supplementary Figure 1 — Composition of fecal microbiota in each sample of the NRDS and non-NRDS groups. (A) Phylum-level composition of fecal microbiota in each sample of both groups. (B) Genus-level composition of fecal microbiota in each sample of both groups. NRDS, neonatal respiratory distress syndrome. [file Image_1.tif]

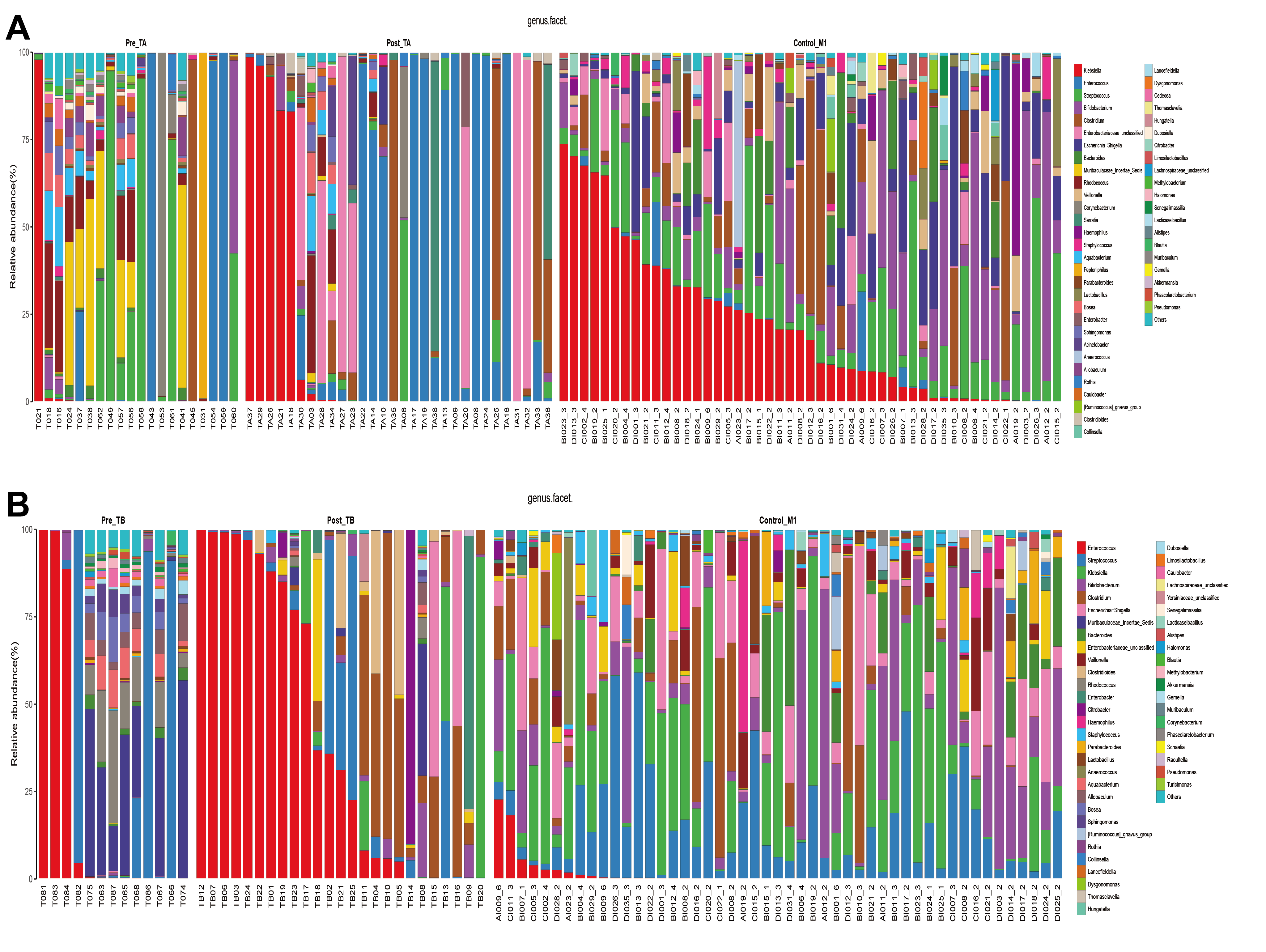

Supplement: Supplementary Figure 2 — Composition of the fecal microbiota in each sample under different treatment methods. (A) Composition of fecal microbiota at the genus level among Pre-TA, Post-TA, and Control-M1 groups. (B) Composition of fecal microbiota at the genus level among Pre-TB, Post-TB, and Control-M1 groups. Pre, pre-treatment; Post, post-treatment; TA, antibiotic-only treatment group; TB, antibiotic plus probiotics treatment group; M1, one month. [file Image_2.tif]

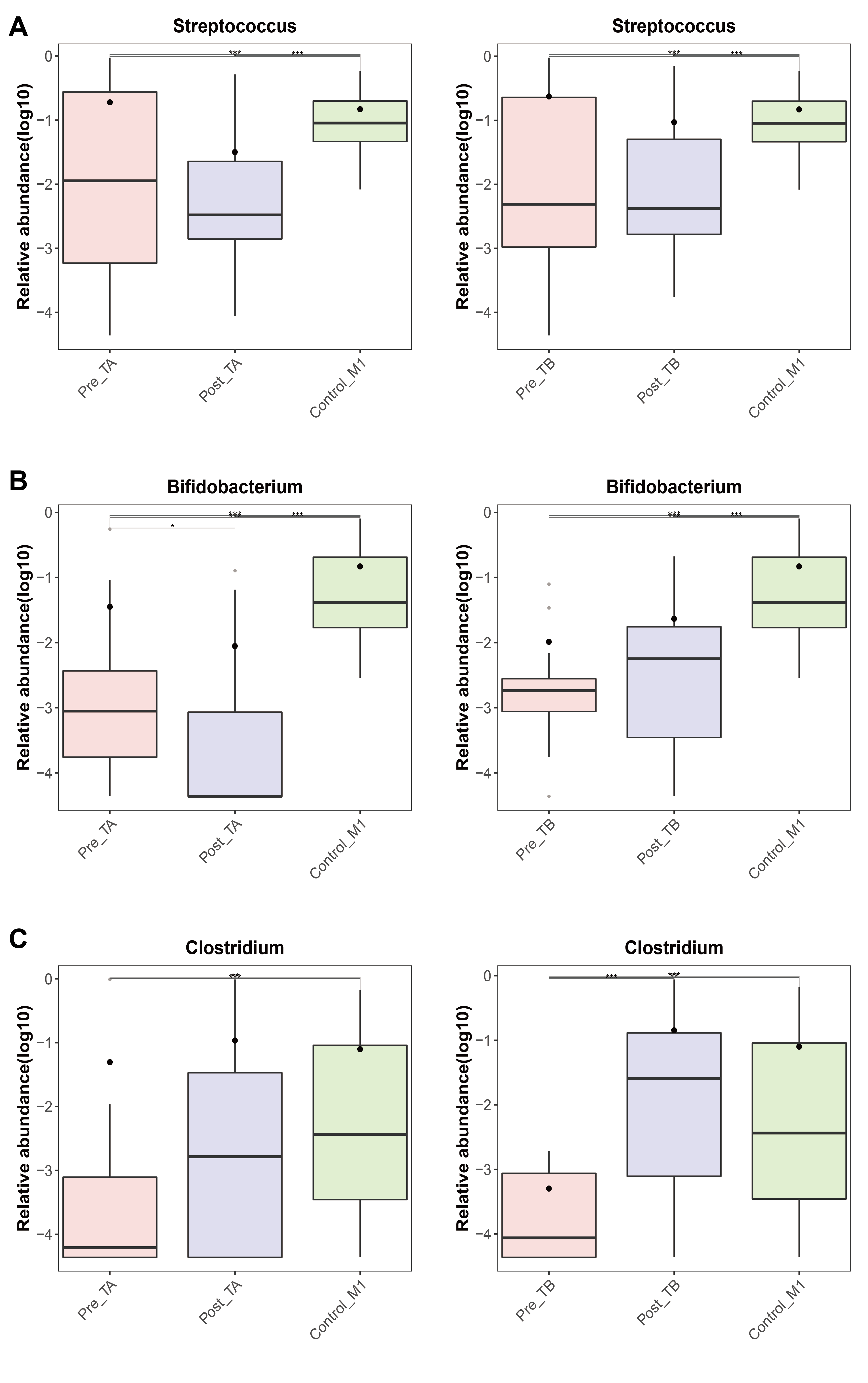

Supplement: Supplementary Figure 3 — Changes in relative abundance of three microbial communities under different treatment modalities. Differences in the relative abundance changes of Streptococcus (A), Bifidobacterium (B), and Clostridium (C) in TA and TB treatment modalities. The relative abundance of each bacteria was represented by the mean ± SE. Significance of the differences in relative abundance was evaluated using Wilcoxon rank-sum tests (*p < 0.05; **p < 0.01; and ***p < 0.001). Pre, pre-treatment; Post, post-treatment; TA, antibiotic-only treatment group; TB, antibiotic plus probiotics treatment group; M1, one month. [file Image_3.tif]
